# A Conserved Hydrophobic Moiety and Helix–Helix Interactions Drive the Self-Assembly of the Incretin Analog Exendin-4

**DOI:** 10.3390/biom11091305

**Published:** 2021-09-03

**Authors:** Martin Wolff, Klaus Gast, Andreas Evers, Michael Kurz, Stefania Pfeiffer-Marek, Anja Schüler, Robert Seckler, Anja Thalhammer

**Affiliations:** 1 Department of Physical Biochemistry, University of Potsdam, D-14476 Potsdam, Germany; martin.wolff@uni-potsdam.de (M.W.); khpgast@uni-potsdam.de (K.G.); schuela@uni-potsdam.de (A.S.); seckler@uni-potsdam.de (R.S.); 2 Sanofi-Aventis Deutschland GmbH, D-65926 Frankfurt, Germany; Andreas.Evers@merckgroup.com (A.E.); Michael.Kurz@sanofi.com (M.K.); Stefania.Pfeiffer-Marek@sanofi.com (S.P.-M.)

**Keywords:** biophysics, diabetes, peptides, oligomerization, conformational change, molecular modeling, static and dynamic light scattering, spectroscopy

## Abstract

Exendin-4 is a pharmaceutical peptide used in the control of insulin secretion. Structural information on exendin-4 and related peptides especially on the level of quaternary structure is scarce. We present the first published association equilibria of exendin-4 directly measured by static and dynamic light scattering. We show that exendin-4 oligomerization is pH dependent and that these oligomers are of low compactness. We relate our experimental results to a structural hypothesis to describe molecular details of exendin-4 oligomers. Discussion of the validity of this hypothesis is based on NMR, circular dichroism and fluorescence spectroscopy, and light scattering data on exendin-4 and a set of exendin-4 derived peptides. The essential forces driving oligomerization of exendin-4 are helix–helix interactions and interactions of a conserved hydrophobic moiety. Our structural hypothesis suggests that key interactions of exendin-4 monomers in the experimentally supported trimer take place between a defined helical segment and a hydrophobic triangle constituted by the Phe22 residues of the three monomeric subunits. Our data rationalize that Val19 might function as an anchor in the N-terminus of the interacting helix-region and that Trp25 is partially shielded in the oligomer by C-terminal amino acids of the same monomer. Our structural hypothesis suggests that the Trp25 residues do not interact with each other, but with C-terminal Pro residues of their own monomers.

## 1. Introduction

Insulin secretion is essentially controlled by the interaction of two incretin hormones, the glucose-dependent insulinotropic polypeptide (GIP) and the glucagon-like peptide-1 (GLP-1) with their receptors GIPR and GLP-1R, respectively. GLP-1 has attracted attention for its use in the treatment of type 2 diabetes. GLP-1 is highly homologous to several other peptide hormones involved in glycemic control and energy metabolism, such as glucagon, GLP-2, oxyntomodulin and GIP. The binding of all these peptides to their receptors, all members of the class B G-protein coupled receptor family, happens in a mainly α-helical conformation in a very similar manner [1]. Another commonality of these peptides is a highly conserved hydrophobic moiety FhxWL (with h denoting any hydrophobic and x denoting any amino acid, respectively) (Figure S1), which has been previously suggested to play a role in the aggregation of glucagon [2].

A major drawback hindering the pharmaceutical use of GLP-1 is its rapid degradation in vivo by the enzyme dipeptidyl-peptidase IV. This has stimulated the search for peptidase resistant analogues. The first stable marketed GLP-1 mimetic was exendin-4, a peptide of 39 AA (amino acid) residues that was originally isolated from the venom of the lizard *Heloderma suspectum*. Exendin-4 shares about 50% sequence identity with GLP-1. Since then, several advanced unimolecular dual or triple agonist peptides targeting more than one of the receptors indicated above have been developed (see [3] for a review). In line with this, we recently characterized the potent dual GLP-1/glucagon agonist Dual-Cex ([4], assigned as peptide 7 in [5]), which was accomplished by engineering sequence sections of oxyntomodulin into exendin-4.

A series of studies published by the Andersen lab in the early 2000s [6,7,8,9,10] provided basic structural information of exendin-4 concerning secondary and tertiary structure. Notably, peptide conformation is highly dependent on solvent conditions. In principle, the amino acids 9–27 of exendin-4 form an α-helix under various solvent conditions, while the nine N-terminal amino acids are essentially unstructured [7]. The superposition of unbound exendin-4 in the presence of 30% trifluoroethanol (TFE) obtained from NMR [10] and receptor-bound exendin-4(9–39) in crystals shows striking similarities [11]. This suggests that the helical structure preferences of exendin-4 in solution are related to its receptor binding affinity. Exendin-4 further harbors a C-terminal segment, which can fold into the so-called tryptophan (Trp-) cage, a particular structural motif, that was clearly identified in aqueous solutions containing TFE [10]. Under these conditions, the C-terminal segment of exendin-4 folds back on the central α-helix and forms what has been designated the smallest stable known protein tertiary structure. Here, the two hydrophobic bulky amino acids Phe22 and Trp25, which are part of the conserved hydrophobic moiety FhxWL, are closely associated with Gly26 and Pro31, Pro37 and Pro38, resulting in the essential enclosure of Trp25 [9]. This fold is only partially populated in aqueous glycol solvent [8] and not formed in the presence of dodecylphosphocholine micelles, where residues 31–39 are unstructured and highly dynamic. Independent of the formation of the actual Trp-cage conformation, a hydrophobic cluster between Trp25 and Pro31 has been suggested to provide a stable capping of the exendin-4 α-helix [8]. To avoid misinterpretation, we will refer to the specific conformation of the C-terminal segment in 30% TFE as the ‘Trp-cage conformation’ and use the term ‘hydrophobic moiety’ when referring to other, less strictly defined conformations of this segment in the following.

Based on the stable Trp-cage conformation of exendin-4 in 30% TFE, a variety of Trp-cage miniproteins have been designed. Most of them were derived from the initial Trp-cage variety TC5b [9], which was mainly realized by N-terminal truncation of the exendin-4 backbone and several point mutations. TC5b represents a non-fluxional, compact, and globular structure in aqueous environments [9]. Trp-cage miniproteins have been the subject of numerous studies concerning solvent interactions, folding processes, stability, and aggregation tendency [12,13,14,15,16], mainly due to their small size being a helpful property in computer simulations. To our knowledge, literature on the oligomeric state of Trp-cage miniproteins is limited to a single study using a cyclized Trp-cage variant [17], facilitating analytical ultracentrifugation (AUC).

Likewise, reports on the oligomerization of exendin-4 are scarce and structural details of the resulting oligomers are not available to date. And yet, self-assembly of pharmaceutical peptides is not only of general basic interest, but is fundamental for biological activity, stability, absorption, and lifetime under formulation conditions. The existence of oligomeric states of exendin-4 in buffer or under formulation conditions and their possible characteristics have been suggested only indirectly from NMR- and CD-spectroscopic data. Also, the state of exendin-4 in aqueous solutions containing TFE has not been directly determined up to now. From NMR measurements of translational diffusion coefficients Wang et al. [18] proposed the existence of dimers or trimers. In aqueous solution, Hudson suggested the oligomerization of exendin-4 via amphipathic helix–helix interactions, yielding helix-bundles [7], which is still a matter of debate.

In contrast to these previous reports, we use combined static and dynamic light scattering (SLS/DLS) for direct determination of the molecular mass and hydrodynamic radius. This enables us to unequivocally and directly determine the association behavior of exendin-4 in different solvent and concentration conditions. We relate our experimental results to a molecular structural hypothesis, which was built on an alignment of exendin-4 with the crystal structure of a cyclized Trp-cage variant [17] to describe molecular details of exendin-4 oligomers. We discuss the validity of this hypothesis based on spectroscopic and light scattering data. We put special focus on the role of the conserved hydrophobic moiety FhxWL in the association of exendin-4, utilizing a monomeric exendin-4 derived peptide Dual-Cex [4,5] and a set of derived mutants. We further exploit differences in the properties of exendin-4 and Dual-Cex to discuss the role of helix–helix interactions in self-assembly.

## 2. Materials and Methods

### 2.1. Sample Preparation

**Figure 1:**
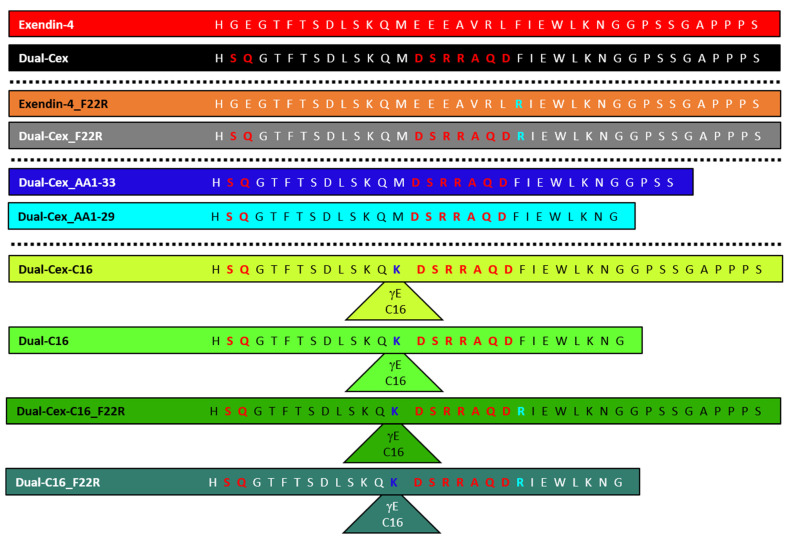
Amino acid sequences of all peptides. Sequence stretches of the dual agonist peptides derived from Glucagon are marked in bold red letters. Substitution of a Phe residue at position 22 by Arg is indicated in bold cyan letters. In the fatty acid-conjugated peptides, Ser in position 2 was replaced by d-Ser and Met at position 14 was replaced by Lys (blue), which was conjugated to γ-glutamic acid followed by a fully saturated palmitic acid chain indicated by triangles.

All peptides (Figure 1) were synthesized and purified as reported previously [5]. PBS was prepared from tablets (Calbiochem). Sodium acetate, glycerol, *m*-cresol, and l-methionine were supplied from Merck. TFE > 99% (GC) was purchased from Fluka. All solvents and solutions were prepared using ultrapure water (ELGA LabWater, Veolia Water Technologies Deutschland GmbH, Celle, Germany) and were filtered and degassed after preparation using 0.45µm Millicup filter units (Millipore). Peptides were dissolved in and dialyzed against the respective solvent at 4 °C overnight (molecular weight cutoff (MWCO) 3500, Spectra/Por, Sigma-Aldrich Chemie GmbH, Taufkirchen, Germany) with one exchange of the dialysis medium. All experiments were either carried out at pH 7.4 in PBS or at pH 4.5 in 43 mM sodium acetate, pH 4.5, 18 mg/mL Glycerol 85%. Peptide concentrations were determined photometrically using their specific absorbance A (280 nm, 1 cm, 1 mg/mL) calculated with the ProtParam tool [19] using the amino acid sequence and the calculated molecular mass of the peptides including the alkyl chain if present as specified in Table 1.

### 2.2. Static and Dynamic Light Scattering

Simultaneous SLS and DLS experiments were performed at a scattering angle of 90° with a custom-built apparatus equipped with a 0.5 W diode-pumped continuous-wave laser (Cobolt Samba 532 nm, Cobolt AB, Solna, Sweden), a high quantum yield avalanche photodiode, and an ALV 7002/USB 25 correlator (ALV GmbH, Langen, Germany) as previously published [4]. All samples were subjected to ultracentrifugation at 60,000 g for 30 min directly prior to the measurements. Briefly, mean scattering intensities I and the time-autocorrelation function (ACF) of the fluctuations in the instantaneous scattering intensity were recorded in intervals of 8 s and averaged over a total of 50 to 500 data pairs. SLS/DLS measurements were done in 3 mm path length microfluorescence cells (105.251-QS, Hellma, Germany) in a Peltier thermostat-controlled cell holder at 23 °C. Apparent molar masses were estimated from the relative scattering intensities using toluene as a reference sample and applying a refractive index increment (dn/dc) = 0.19 mL/g. The translational diffusion coefficients D were obtained from the measured autocorrelation functions using the program CONTIN [20]. The diffusion coefficients were converted into R_S_ via the Stokes–Einstein equation R_S_ = k_B_T/(6πηD), where k_B_ is Boltzmann’s constant, T is the temperature in Kelvin, and η is the solvent viscosity.

### 2.3. CD Spectroscopy

CD spectra in the far- and near-UV region were recorded in a Jasco J-715 or a Jasco J-815 spectropolarimeter equipped with a Peltier thermostat-controlled cell holder (Jasco, Pfungstadt, Germany) using quartz cuvettes with appropriate path lengths between 0.1 mm and 10 mm (Hellma, Müllheim, Germany). All samples were centrifuged with at least 12,000× *g* for 15 min directly prior to the measurements. After baseline correction, ellipticities θ from far-UV CD measurements were converted into mean-residue ellipticities θ_MRW_ using the respective mean-residue weight of each peptide. α-helix content was estimated from θ_MRW_ at 222 nm using an empirical equation [21]. The near-UV CD spectra were converted to molar ellipticities θ_M_ because of the identical number of aromatic chromophores of all peptides.

### 2.4. Trp Fluorescence Spectroscopy

Trp fluorescence spectra were recorded in the wavelength range from 290 to 450 nm on an FP-8500 spectrometer (Jasco, Pfungstadt, Germany) using an excitation wavelength of 280 nm at 23 °C in a 3 mm path length microfluorescence cell (105.251-QS, Hellma, Müllheim, Germany). Spectra were baseline-corrected and normalized to peptide concentration. In addition, spectra of l-tryptophan collected in the different solvents were used for normalization to eliminate weak contributions of the solvent to quantify quenching effects, if necessary. Fluorescence spectra recorded in a concentration range impacted by the inner filter effect were normalized to their fluorescence maxima to avoid overinterpretation of fluorescence intensity information.

### 2.5. NMR Spectroscopy

NMR spectra of Dual-Cex were recorded on a Bruker AVANCE-NEO 700 spectro-meter (Bruker, Billerica, MA, USA) operating at a proton frequency of 700.13 MHz. The instrument was equipped with a 5 mm TCI cryoprobe. Spectra of Dual-Cex were recorded in a mixture of 70% H_2_O/D_2_O (9:1), 100 mM acetate buffer, pH 4.6 and 30% d3-TFE (euriso-top, 99.0% D) at 310 K and in H_2_O/D_2_O (9:1), 100 mM acetate buffer, pH 4.6 at 310 K. Based on the analysis of several 2D-spectra including DQF-COSY, TOCSY, NOESY, ^1^H,^15^N-HSQC and ^1^H,^13^C-HSQC a complete assignment of ^1^H-chemical shifts was achieved (Tables S1 and S2).

## 3. Results

### 3.1. The Experimentally Determined Preferential Formation of Exendin-4 Trimers Suggests a Structural Hypothesis Based on Molecular Modelling

**Figure 2:**
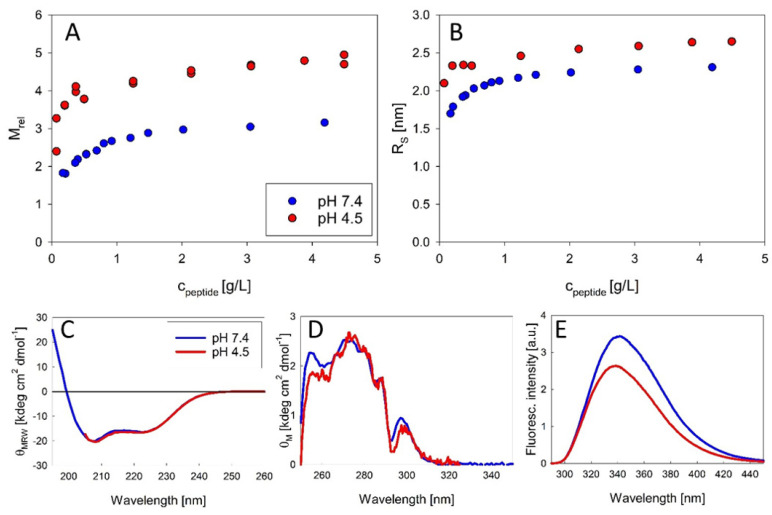
Structural parameters of exendin-4 at pH 7.4 and pH 4.5. (**A**,**B**) show association equilibria in the context of relative molecular masses M_rel_ (**A**) and Stokes radii R_S_ (**B**) measured at 23 °C. Far-UV CD (**C**), near-UV CD (**D**) and Trp fluorescence (**E**) spectra were measured at peptide concentrations > 0.1 mM, indicative of exendin-4 in the oligomeric state and fluorescence intensity is shown normalized to peptide concentration. Light scattering data for exendin-4 at pH 7.4 have been published previously [4].

The molecular masses of exendin-4 were assessed by static light scattering (Figure 2A) at physiological and formulation pH (pH 7.4 and pH 4.5, respectively). Exendin-4 forms on average trimers at neutral pH at sufficiently high peptide concentrations. At acidic pH, exendin-4 forms oligomers of higher molecular mass, probably by an additional population of dimers of trimers. Dissociation is obvious at peptide concentrations below 0.2 mM at both pH regimes from a decrease of both relative mass and R_S,_ determined by DLS (Figure 2B). The association of the trimeric subunits to the dimer of trimers does not impact peptide conformation on the secondary structure level, as obvious from the identical far-UV CD spectra at both pH regimes (Figure 2C). It also has no impact on the conformation of the hydrophobic moiety, as near-UV CD spectra are essentially identical at pH 4.5 and pH 7.4 (Figure 2D) and the maximum wavelength from fluorescence spectra (Figure 2E) is similar. The minor differences in the near-UV and fluorescence spectra likely reflect solvent effects.

**Figure 3:**
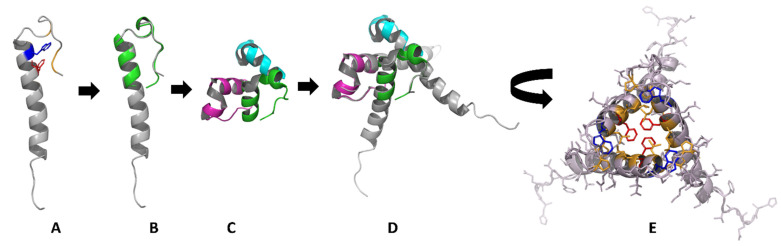
Structural hypothesis on the self-assembly of exendin-4. (**A**) NMR structure of exendin-4 in 30% TFE (PDB 1JRJ) with the conserved hydrophobic domain shown in colors (red Phe22, blue Trp25, orange Pro31, Pro37, Pro38). (**B**) Alignment of exendin-4 (gray) with the X-ray structure of a monomeric subunit of the cyclized Trp-cage miniprotein (PDB 3UC7, green). (**C**) Smallest oligomeric subunit of the cyclized Trp-cage miniprotein found in the crystals is a trimer (Trp-cage miniprotein subunits in the trimer are shown in green, pink and light blue). (**D**) Alignment of exendin-4 monomers (gray) with the trimeric subunit of the cyclized Trp-cage miniprotein (magenta, green and cyan). (**E**) Removal of the cyclized Trp-cage miniprotein trimer and planar projection visualize a structural hypothesis on the molecular nature of an exendin-4 trimer with the hydrophobic domain buried in the oligomer interior (red Phe22, blue Trp25, orange other hydrophobic amino acids).

Justified by the experimentally verified formation of exendin-4 trimers, we used molecular modelling to establish a structural hypothesis in order to grasp molecular details of the according assemblies (Figure 3). The Trp-cage miniprotein TC5b was developed in earlier studies [9] by successive truncation of the native exendin-4 N-terminus. Crystallization of a cyclic Trp-cage variant resulted in dimers of trimers [17]. The NMR structure of exendin-4 in 30% TFE (PDB 1JRJ, Figure 3A) aligns well with the monomeric subunit of the cyclized Trp-cage miniprotein (Figure 3B). Alignment of the exendin-4 structure onto the trimeric subunit of the crystallized Trp-cage miniprotein (Figure 3C) with the Pymol (The PyMOL Molecular Graphics System, Version 2.0 Schrödinger, LLC, Mannheim, Germany) align function results in a trimeric exendin-4 model (Figure 3D). In this alignment, the hydrophobic moiety is buried within the oligomer interior with Phe22 as one of the key interaction partners between the monomers. In addition, the model suggests the interaction of helical segments (Figure 3E). R_S_ calculated from the exendin-4 trimer model using HYDROPRO with a v̅ of 0.725 cm^3^/g [22] resulted in 2.2–2.4 nm, which is in line with the experimentally determined R_S_ at exendin-4 concentrations representing the oligomeric peptide. Such 3D-alignment of exendin-4 on the full hexameric crystal form of the Trp-cage miniprotein would also provide a reasonable hexameric model as a dimer of trimers for exendin-4 (alignment not shown) in line with our light scattering data at acidic pH.

Due to the high flexibility of the N-terminal tail, it is not possible to confirm the molecular nature of the quaternary structure of exendin-4 by a high-resolution crystal structure. Therefore, we used spectroscopy and light scattering techniques to investigate the validity of this structural hypothesis.

### 3.2. The Contribution of Helix–Helix Interaction in Self-Assembly of Exendin-4 Is Evident from the Comparison of Exendin-4 and Dual-Cex

**Figure 4:**
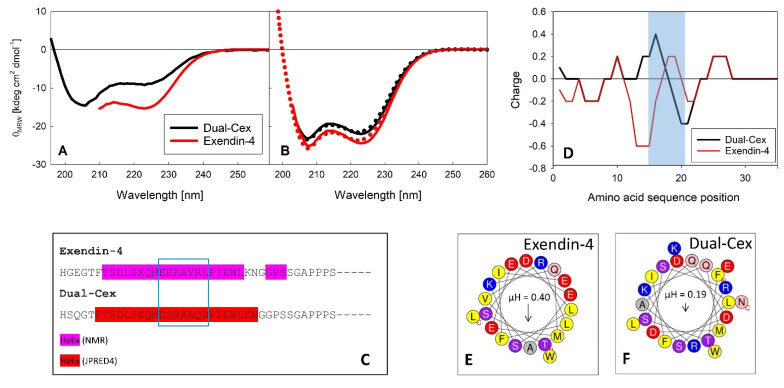
Secondary structure of exendin-4 and Dual-Cex. Far-UV CD spectra of exendin-4 and Dual-Cex were measured in PBS (**A**) and in 30% TFE (**B**) at pH 7.4 at concentrations < 0.1 mM which grant a monomeric state of all peptides. Amino acid sequence alignment of exendin-4 and Dual-Cex. (**C**) Amino acids in an α-helical conformation as derived from NMR experiments or predictions (JPRED4 [23]) are colored pink and red, respectively. A charge plot of exendin-4 and Dual-Cex calculated with the Emboss charge tool [24], using a sliding window of five, is shown in (**D**). The blue frames in (**C**) and (**D**) indicate the main difference between both peptides originating from the sequence stretch engineered into the exendin-4 backbone from oxyntomodulin in Dual-Cex. Helical wheel projections of exendin-4 (**E**) and Dual-Cex (**F**) using amino acids 6–28 comprising the central helical part of both peptides were calculated with HeliQuest [25]. Hydrophobic moments (µH) are indicated.

While exendin-4 self-assembles into an average trimeric structure at neutral pH, its derivative Dual-Cex is essentially monomeric, as reported previously ([4] and Figure 6). A comparison of the far-UV CD spectra of exendin-4 and Dual-Cex (Figure 4A,B) shows that the exendin-4 monomer has considerably more secondary structure in aqueous buffer than Dual-Cex. The spectral shape indicates mainly α-helical structure, about 43% in exendin-4 and 22% in Dual-Cex, which is in line with the literature in the case of exendin-4 [7]. In the presence of 30% TFE, the α-helix ratio of both peptides substantially increases to 65–70%, with Dual-Cex being only about 8% less helical than exendin-4. It is worthy to discuss these differences in the helicity of exendin-4 and Dual-Cex, which necessarily arise from the replacement of a stretch of seven amino acids from exendin-4 by a sequence stretch of similar length from oxyntomodulin in the central region of Dual-Cex (Figure 4C). Comparing the sequences constituting the helical regions of both peptides shows major consequences of the amino acid substitution on peptide charge (Figure 4D) and hydrophobic moment, the latter reporting on the amphipathic character of an α-helix (Figure 4E,F). The hydrophobic moment is severely reduced in Dual-Cex and might explain the different self-assembly tendencies of both peptides. It is thus conceivable, that the oligomerization of exendin-4 necessitates hydrophobic interactions between the amphipathic α-helical segments. Comparable hydrophobic interactions are not established among Dual-Cex monomers, which is markedly less helical in buffer and the helix, if established, is considerably less amphipathic. Taken together, this hinders self-assembly of Dual-Cex.

### 3.3. The Hydrophobic Moiety Participates in the Self-Assembly of Exendin-4

**Figure 5:**
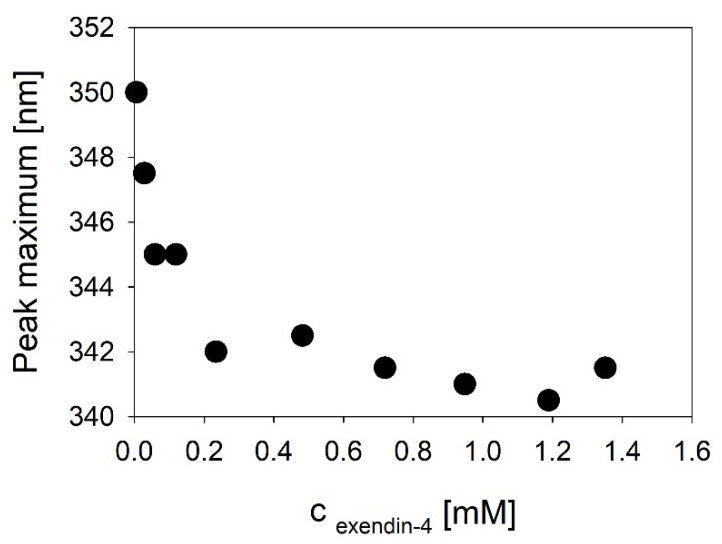
Spectral signature changes of the hydrophobic moiety accompanying self-assembly of exendin-4 at pH 7.4. Wavelength of the maximum Trp fluorescence spectra recorded after excitation at 280 nm in PBS, pH 7.4 was plotted as a function of exendin-4 concentration.

Concentration-dependent measurements of Trp fluorescence indicate that self-assembly of exendin-4 into trimers is accompanied by either shielding or conformational changes of the conserved hydrophobic moiety. This is expressed in a reduced solvent exposure reported by a shift of the fluorescence emission maximum to lower wavelengths (Figure 5).

**Figure 6:**
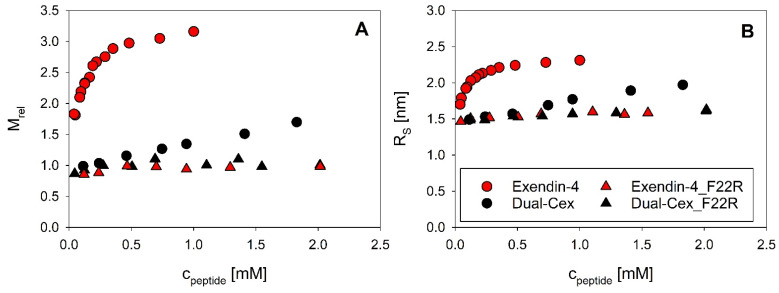
Association equilibria of exendin-4 and Dual-Cex and the F22R mutants of both peptides. Relative molecular masses M_rel_ (**A**) and Stokes radii R_S_ (**B**) at pH 7.4 were measured at 23 °C. Data for exendin-4 and Dual-Cex have been published previously [4].

This is an interesting finding, as our structural hypothesis indicates that the conserved hydrophobic moiety is part of the oligomer interface. Therefore, we designed mutants of exendin-4 and Dual-Cex, in which we disturbed this hydrophobic moiety by exchanging the hydrophobic Phe22 by a positively charged Arg (F22R). Strikingly, this mutation results in full inhibition of the self-assembly of exendin-4, as the resulting exendin-4_F22R is monomeric over a wide concentration range. In the case of the monomeric Dual-Cex, M_rel_ and R_S_ show a minor increase over a concentration range comprising two orders of magnitude, which is likely due to intermolecular attraction between peptide monomers. Introduction of the F22R mutation in Dual-Cex (Dual-Cex_F22R) affects these intermolecular interactions, as obvious from a lack of the narrow slope in both concentration-dependent M_rel_ and R_S_ (Figure 6). The F22R mutation has only a minor impact on the secondary structure of both peptides (Figure S3).

### 3.4. Successive Exposure of the Hydrophobic Moiety in the Monomeric Dual-Cex Powers Association

**Figure 7:**
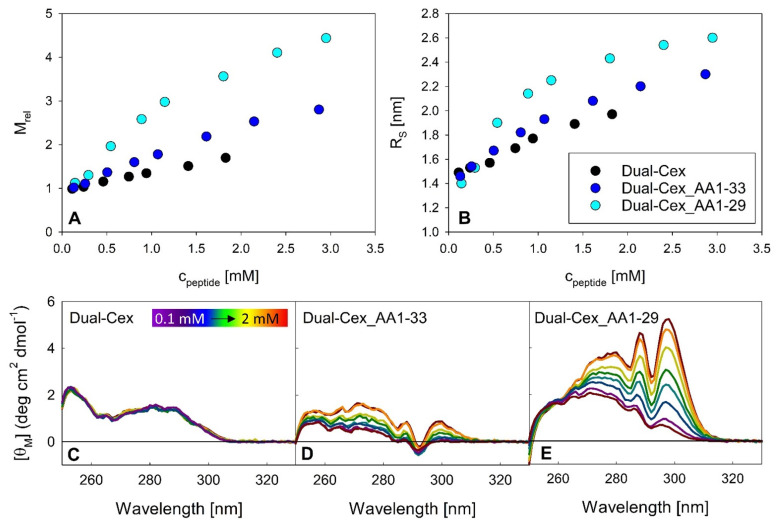
Structural consequences of C-terminal truncation of Dual-Cex. (**A**,**B**) show association equilibria in the context of relative molecular masses M_rel_ (**A**) and Stokes radii R_S_ (**B**) of Dual-Cex and two C-terminally truncated Dual-Cex variants measured at 23 °C. Near-UV CD spectra (**C**–**E**) were measured for a peptide concentration range from around 0.1–2 mM.

In the Trp-cage miniprotein, the C-terminus folds back towards the N-terminal part of the peptide and thus shields the amino acids constituting the hydrophobic moiety, Phe22 and Trp25 [10]. This is also the case for exendin-4 in 30% TFE and to some extent also in aqueous buffer. However, exendin-4 is not an ideal candidate to address if such a shielding modulates oligomerization, as it is in an oligomeric state per se. Consequently, it would provide substantial interfering background signal. We therefore utilized the monomeric nature of Dual-Cex and designed mutants which are restricted in shielding the hydrophobic moiety by successive truncation of the C-terminus. Dual-Cex_1-33 lacks the six C-terminal residues and Dual-Cex_1-29 the ten C-terminal residues, respectively. Figure 7A,B show association equilibria of Dual-Cex and the respective mutants. Interestingly, successive truncation of the C-terminus, and thus reduced shielding of the hydrophobic moiety, results in oligomeric structures. Thereby, truncation of the C-terminus by six residues yields lower molecular weight assemblies compared to truncation by ten residues. It is important to note that oligomerization proceeds over an unusually wide range of peptide concentrations in both mutants and does not reach a stable plateau even at the highest measured concentrations. This points towards the idea that oligomerization can be enforced by the provision of exposed hydrophobic surfaces. At the same time, the resulting oligomers do not seem to constitute defined structures but rather present hydrophobic interfaces to the surrounding solvent which are competent for the incorporation of further peptide monomers. As obvious from near-UV CD spectra (Figure 7C–E), the Trp environment of the C-terminally truncated mutants is strongly responsive to oligomerization, especially in the case of Dual-Cex_AA1-29, indicating a major conformational rearrangement of the hydrophobic moiety in the peptide oligomers.

### 3.5. Fatty Acid Driven Self-Assembly of Dual-Cex and Assembly Stability Is Modulated by Modification of the Hydrophobic Moiety

**Figure 8:**
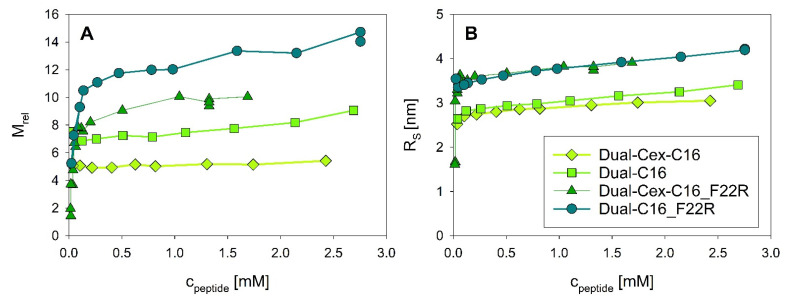
Association equilibria of palmitic acid conjugated Dual-Cex and derived variants. Relative molecular masses M_rel_ (**A**) and Stokes radii R_S_ (**B**) were measured at 23 °C in PBS at pH 7.4. The according data for Dual-Cex-C16 and Dual-C16 were previously published [4] and are shown for contextual reasons.

We have previously shown that self-assembly of Dual-Cex is strongly driven by the conjugation of fatty acids of increasing length [4]. Here, we utilize this knowledge to enforce the self-assembly of Dual-Cex and the derived mutants by conjugation of a palmitic acid (Dual-Cex-C16). As previously shown, palmitic acid fusion to Dual-Cex results in, on average, pentameric assemblies, which are highly stable towards dilution in the concentration range accessible by light scattering [4]. All palmitic acid conjugated mutants of Dual-Cex self-assemble, driven by the hydrophobic fatty acyl chain. However, major mutation-dependent differences in the nature and stability of the assemblies are obvious. These give additional insights into the role of the conserved hydrophobic moiety for oligomerization of Dual-Cex (Figure 8). Relaxation of the hydrophobic moiety by substitution of Phe22 with Arg (Dual-Cex-C16_F22R) leads to the formation of oligomers composed of more, on average ten, monomeric subunits and larger dimensions in terms of R_S_. These self-assemblies are considerably less stable towards dilution, compared to Dual-Cex-C16, underlining the drastic consequences of the F22R substitution on oligomerization of the fatty acyl-free mutant Dual-Cex_F22R. C-terminal truncation of Dual-Cex-C16 by ten amino acids, resulting in full exposure of the hydrophobic moiety (Dual-C16) also results in assemblies comprising more monomeric subunits, on average eight. In this case, R_S_ is not affected, which is likely due to the smaller size of the respective monomers. In contrast to the F22R substitution, C-terminal truncation does not decrease dilution stability in the concentration range accessible by light scattering, in line with the amplified oligomerization of the respective fatty acid-free C-terminally truncated mutant Dual-Cex_AA1-29. Finally, the combination of both mutations, F22R substitution as well as C-terminal truncation, in a palmitic acid conjugated Dual-Cex backbone (Dual-C16-F22R) even further increases the size of the self-assemblies in terms of the number of monomeric subunits. The susceptibility of these assemblies towards dilution is high, apparently an effect transported from the F22R mutation.

### 3.6. The Defined Trp-Cage of the Peptide Monomer Cannot Be Presumed in Aqueous Buffer

**Figure 9:**
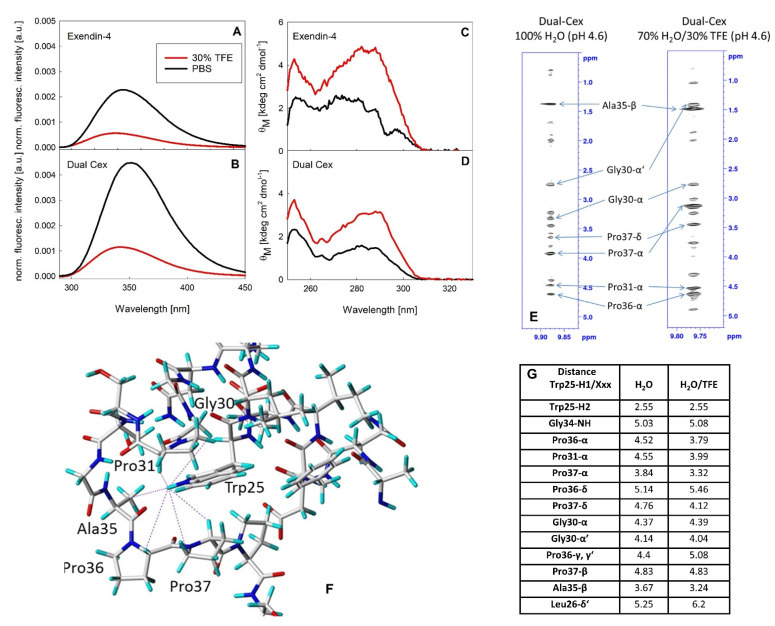
Spectral signatures of exendin-4 and Dual-Cex in PBS and 30% TFE. Normalized Trp fluorescence (**A**,**B**) and near-UV CD (**C**,**D**) spectra of exendin-4 (**A**,**C**) and Dual-Cex (**B**,**D**) in PBS and PBS + 30% TFE. Regions of the NOESY spectra of Dual-Cex in water and in 30% TFE illustrating NOEs between Trp25-H1 and protons of residues involved in the formation of the Trp-cage (Gly30, Pro31, Ala35, Pro36, Pro37) (**E**). Interproton distances (illustrated as dashed lines in (**F**); grey, red, blue and light blue colors represent carbon, oxygen, nitrogen and hydrogen atoms, respectively) were obtained by integration of the NOESY peaks using the distance between Trp25-H1 and Trp25-H2 as reference peak (distance was set to 2.55 Å) (**G**).

The Trp-cage miniprotein is characterized by a stable tertiary fold in aqueous buffer, evidenced by different spectroscopic techniques [9]. As our structural model utilizes the structure of the exendin-4 monomer in 30% TFE, it is important to clearly distinguish between the conformations of the hydrophobic moiety in this environment and in aqueous buffer. A stably folded conformation analogous to that of the Trp-cage miniprotein can be induced by addition of TFE in exendin-4 and Dual-Cex (Figure 9 and [5]), as demonstrated by a shift of the maximum Trp fluorescence to around 340 nm, indicating that Trp25 is not fully solvent exposed. Similarly, the ellipticities around 290 nm in the near-UV CD spectra show a substantial signal in the presence of 30% TFE, indicating a considerable degree of rigidity of the Trp environment. Interestingly, a stable fold similar to that of the Trp-cage miniprotein is not evident for exendin-4 and the derived Dual-Cex in a fully aqueous environment. In fact, the hydrophobic moieties of both peptides show a similar yet different spectroscopic fingerprint of the environment around Trp25 in aqueous buffer (Figure 9). Trp fluorescence spectra show a maximum around 350 nm, indicating an essentially full solvent exposure of Trp25. Likewise, near-UV CD spectra exhibit low signal amplitudes around 290 nm, demonstrating a high degree of flexibility of Trp25. Due to the oligomeric nature of exendin-4 and the corresponding poor quality of NMR spectra in water, NMR spectra were exclusively measured for the monomeric Dual-Cex in water and in 30% TFE (Figure 9E). Interproton distances between Trp25-H1 and residues of the conserved hydrophobic moiety and C-terminus, which are known to contribute to Trp-cage formation (Figure 9G) were determined from the NOESY spectra. Although long range correlations to the same residues are obtained (Leu26, Gly30, Pro31, Gly34, Ala35, Pro36, Pro37), differences for some of the distances and clearly different highfield shifts for Pro37-Hα and Gly30- Hα‘ indicate a slightly different geometry of the Trp-cage in both solvents.

### 3.7. Exendin-4 Oligomers Are Not Very Compact and Highly Dynamic

**Figure 10:**
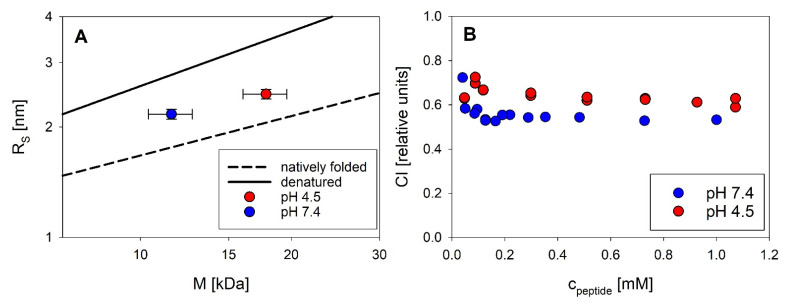
Compactness of exendin-4 at pH 7.4 (blue) and pH 4.5 (red). Compactness of ~0.3 mM exendin-4 at 23 °C, visualized using scaling laws of the type R_S_ = aM^b^ with a scaling exponent b of 0.33 for globular and of 0.5 for denatured proteins, respectively. Error bars indicate average experimental errors of 10 and 3% for the apparent molecular mass and R_S_, respectively. Reference data sets reporting on the average compactness of natively folded (dashed line) and denatured (solid line) proteins are shown for comparison (**A**). CI were calculated as compactness relative to the scaling behavior of unfolded and folded reference proteins, assuming CI of 0 and 1, respectively (**B**).

Our spectroscopic data in buffer underline the flexible and partially unfolded character of exendin-4 reported in the literature [10]. We were interested to see if this translates to the exendin-4 oligomers. Monitoring peptide compactness in a relative context is possible by comparing the hydrodynamic dimensions of a peptide of known molecular mass against the scaling behavior of large sets of well-folded and denatured proteins (Figure 10A). Concentration-dependent changes in oligomeric dimensions can be directly followed by the compactness index (CI, Figure 10B), which we introduced in [4]. CI of exendin-4 were calculated from relative masses and R_S_ (Figure 10A,B) in a concentration-dependent manner. At pH 7.4, the CI of exendin-4 report an intermediate compactness of the peptide halfway between globular folded (CI = 1) and denatured (CI = 0) proteins. The association into dimers of trimers at pH 4.5 results in a slightly increased compaction, as obvious from the increase in CI. Self-assembly of exendin-4 is not accompanied by peptide compaction, as obvious from constant CI over the concentration range relevant for self-assembly. This indicates a flexible and dynamic nature of exendin-4 oligomers, but of course does not allow any statements on the flexibility of the monomeric units in the exendin-4 oligomer on the atomistic level.

## 4. Discussion

A first important matter of this work is a detailed characterization of the association state of exendin-4 under appropriate solution conditions. Up to now, the association state of exendin-4 has not been well defined and controversial opinions about the appearance of oligomerization, the type of oligomers, and the mechanisms of formation have been published [6,7,18]. Hudson and Andersen [6,7] concluded the existence of oligomeric species in strictly aqueous media from the influence of concentration on NMR and CD data. Wang et al. [18] tried to estimate the size of putative oligomers from NMR diffusion-ordered spectroscopy (DOSY), suggesting the formation of trimers. From our SLS data we can directly calculate the mass and thus the number of associated peptide molecules without any assumptions about the arrangement of monomers. Our study therefore provides the first published association equilibria of exendin-4 in aqueous solutions. The oligomeric state of exendin-4 is clearly pH dependent. At pH 7.4, we find an apparently unimodal association into trimers, which is observed over a sufficiently wide concentration range (Figure 2A,B). Therefore, our data substantially add to the knowledge on the association of exendin-4 available from previous studies. The postulation of a trimeric main species is supported by published NMR data, which suggest that a defined oligomeric species is populated over a wide range of concentrations [7]. At pH 4.5, close to the isoelectric point of exendin-4 (pI = 4.86 [26]), oligomer formation is biphasic with the formation of trimers being reinforced, as the trimeric state is already approached at lower peptide concentrations compared with pH 7.4. With increasing concentration, larger oligomers, likely a mixture of trimers and dimers of trimers are obvious. This differential oligomerization behavior is attributable to a different degree of electrostatic interactions. At both pH regimes, dissociation becomes evident at low concentrations, but complete dissociation into monomers could not be verified, since it likely happens below the detection limit of SLS. The conformation of monomeric subunits regarding the secondary and tertiary structure level is comparable at both pH regimes (Figure 2C–E). The conserved hydrophobic moiety is essentially covered from solvent during trimer formation (Figure 5).

These findings on the global solution structure of exendin-4 stimulated us to establish a structural hypothesis of the smallest oligomer of exendin-4, the trimer at pH 7.4, using molecular modelling based on the crystal structure of an exendin-4 derived Trp-cage miniprotein (Figure 3). Importantly, this hypothesis is fully compatible with all so far presented experimental data and moreover supported by comparing calculated and measured R_S_. In the following, we will facilitate experimental data of selected exendin-4 derived peptides in order to discuss the validity of molecular details of the association of exendin-4 as suggested by this hypothesis. We will restrict this discussion to the smallest oligomer at pH 7.4, but a dimer of trimers at low pH, relevant in the pharmaceutical formulation of exendin-4, may also in principle be rationalized following this hypothesis.

A key structural element of exendin-4 is a central amphipathic α-helix, which has been found in solution [10] and also in the crystal structure of N-terminally truncated exendin-4(9–39) bound to the isolated extracellular domain of human GLP-1R [11]. Our structural hypothesis suggests an interaction of monomeric subunits of exendin-4 in the trimer via helix–helix contacts of the C-terminal half of this helix. Similarly, Hudson et al. concluded an association of exendin-4 via interhelical interaction based on the extensive broadening of NMR resonances in the Gln13 to Leu26 stretch [7]. Interestingly, precisely these amino acids are situated in the oligomer interface in our structural hypothesis and will be denoted as helical interface segments in the following. The high importance of helix–helix interactions of this helical interface segment for association of exendin-4 is directly evidenced by the largely monomeric nature of the exendin-4 derived Dual-Cex, which carries modifications within the respective segment. These modifications encompass Asp15 to Asp21 and apparently heavily impact charge and hydrophobic moment (Figure 4D–F). It is thus conceivable, that the oligomerization of exendin-4 necessitates hydrophobic interactions between the amphipathic α-helical segments. Comparable hydrophobic interactions are not established among Dual-Cex monomers, which is markedly less α-helical in buffer and the α-helix, if established, is considerably less amphipathic. Taken together, this hinders self-assembly of Dual-Cex. Hudson initially suggested a helix-bundle or even coiled-coil state for exendin-4 oligomers. This idea was based on the lack of an isodichroic point in the CD spectra of exendin-4 during melting and a comparatively high melting temperature [7]. The classical picture of a helix-bundle is associated with knobs-into-holes packing of hydrophobic residues of adjacent helices as originally introduced by Crick [27]. This often results in almost parallel or antiparallel orientation of the interacting helices, which are frequently characterized by conserved patterns of amino acids [27]. However, the actual angle of interacting helices constituting a helix-bundle can be relatively wide [28]. As both diagnostics suggested for a helix bundle state by Hudson [7] would also apply for a rather triangular orientation of the helical segments situated in the interface of a trimer architecture, our structural hypothesis is not in conflict with the data presented by Hudson [7]. However, a typical coiled-coil structure of exendin-4 oligomers is not supported by our data. This is because the rather defined oligomers of exendin-4 in aqueous solution are fairly extended, which points towards a certain degree of structural flexibility (Figure 10), with the pH 7.4 trimers being slightly more extended than the dimers of trimers observed at pH 4.5. In contrast to the quite extended nature of exendin-4 oligomers, coiled-coils should be rather compact structures. In addition, we don’t have indications for coiled-coil structures from the measured CD spectra. Typical coiled-coils have CD spectra differing from that of α-helical structures by a stronger negative ellipticity at 222 nm leading to θ_222_/θ_208_ ratios greater than 1 [29]. Furthermore, coiled-coils are rather stable structures that unfold in a cooperative way [30]. This was not observed for exendin-4 at concentrations corresponding to oligomers ([6] and Figure S4).

The C-terminal part of the exendin-4 helix is constituted by the hydrophobic moiety FhxWL comprising AA 22–26, which is highly conserved among related peptides. Thus, the general statement of helix–helix interactions in the helical interface segment (AA13–26) can be partially specified as interactions of this conserved hydrophobic moiety, which are evident in the exendin-4 trimer. This can be derived from progressive shielding of Trp25 going along with oligomerization (Figure 5). Actual evidence for the importance of interactions of this conserved hydrophobic moiety for oligomerization is impressively evidenced by the F22R substituted isoforms of exendin-4 (exendin-4_F22R) and Dual-Cex (Dual-Cex_F22R), in which oligomerization is completely disabled. This accounts for both the trimerization of exendin-4 as well as the marginal association of Dual-Cex and might be enhanced by electrostatic repulsion between the introduced Arg residues. Therefore, it is clear that the conserved hydrophobic moiety is not only interacting in the trimer, but also presents an important driving force for oligomerization. This argues in favor of our structural hypothesis, which suggests strong hydrophobic attraction between the F22 residues of all monomeric subunits in the center of the trimer. It is important to note that the F22R mutation has only a minor impact on helix stability, which clearly allows us to link the loss of oligomerizability of exendin-4_F22R with the specific absence of Phe22 rather than an overall reduction in helicity. Interestingly, Dual-Cex, which is modified in AA15–21, harbors the conserved hydrophobic moiety without any modifications and is essentially monomeric, indicating that interactions of the conserved hydrophobic moieties alone are not sufficient for oligomerization. This proves the essential role of further helix–helix interactions in the oligomerization of exendin-4 and can be rationalized by our structural hypothesis, which suggests close contact and thus hydrophobic interaction of Trp25 with Val19. The latter is replaced by a less hydrophobic Ala in Dual-Cex, which is a possible explanation for the lack of oligomerization of Dual-Cex. This argues for the suggested triangular oligomer architecture of exendin-4 with Val19 functioning as an anchor for neighboring exendin-4 subunits in the trimer.

Our structural hypothesis facilitates an NMR derived monomer structure of exendin-4 in 30% TFE. The impact of TFE on the association state of exendin-4 has not been finally resolved to date. Wang et al. [18] have tried to estimate the size of putative oligomers from NMR DOSY, but the results in the presence of TFE (dimers or trimers) are questionable. Our SLS data (Figure S2A) unequivocally show that exendin-4 has reached a fully monomeric state already at a TFE concentration of 20%. The hydrodynamic dimensions of the monomers are rather large, typical of extended proteins (Figure S5). The absolute ratio of helicity estimated from our CD spectra in 30% TFE is similar to the one of the NMR derived monomer structure, accounting for about 70%. It is obvious that in 30% TFE, exendin-4 has about 20% more α-helical structure than in aqueous buffer (Figure 4B). It is reasonable to assume that this is due to a more elongated conformation of monomers mainly brought about by the central helical part which is stabilized in the presence of TFE. TFE has been described as accumulating near peptide surfaces and to preferentially interact with amino acid side chains [31], therefore under these conditions, the helix can be regarded as essentially covered with TFE molecules. In this perfectly covered TFE-state, exendin-4 is not able to form oligomers. The superior helicity in 30% TFE and the fact that the specific Trp-cage conformation of the C-terminus of exendin-4 is formed under these conditions, but not in aqueous buffer, challenges the suitability of the NMR derived structure of the exendin-4 monomer in 30% TFE as a building block for our molecular modelling approach. Our structural hypothesis specifically relies on the stability of the C-terminal half of the α-helix of exendin-4 in aqueous buffer. A hydrophobic cluster between Trp25 and Pro31, which forms independently of the Trp-cage already in aqueous buffer, has been suggested to provide a stable C-terminal capping of the exendin-4 α-helix previously [7]. Therefore, it is conceivable that TFE stabilizes the helix towards the N-terminal part of the peptide, which is not involved in inter-monomer interaction in our exendin-4 trimer model. The addition of 30% TFE to solutions of exendin-4 and Dual-Cex results in a substantial quenching of Trp fluorescence and an increase in molar ellipticity around 290 nm, indicating the shielding of Trp25 from solvent and a concomitantly reduced flexibility. Trp fluorescence quenching has been recognized as a diagnostic marker for formation of the Trp-cage [32,33]. Therefore, our data clearly evidence the formation of the Trp-cage in the presence of TFE for Dual-Cex and exendin-4 (Figure 9) but cannot resolve to what degree the Trp-cage exists in strictly aqueous media. A direct comparison of the structures of the exendin-4 monomer in water and 30% TFE in single-residue resolution has been previously hampered by the oligomeric nature of exendin-4 and the concomitantly poor-quality NMR spectra [7]. Encouraged by the similar signal changes in the fluorescence and near-UV CD spectra of exendin-4 and Dual-Cex, we took advantage of the monomeric nature of Dual-Cex and recorded NOESY spectra in aqueous buffer and 30% TFE (Figure 9E–G). For exendin-4, formation of the Trp-cage in 30% TFE leads to large highfield-shifts for Gly30-Hα2, Pro37-Hα, and Pro38-CδH_2_ [7]. In the case of Dual-Cex, the addition of 30% TFE induces an equivalent highfield-shift of these protons (Table S1), thus confirming the same Trp-cage conformation for both peptides. However, in the absence of TFE clearly smaller highfield-shifts and some differences in the interproton distances are obtained (Figure 9G and Table S2). This indicates a slightly different geometry of the residues surrounding the side chain of Trp25 and thus a different conformation of the C-terminus and the conserved hydrophobic moiety. It is conceivable that in aqueous buffer the Trp-cage conformation is either transiently sampled, leading to a more open conformation, or represents an alternative defined fold characterized by a different local energy minimum. This interesting question should be addressed by time-resolved structure analytics in future studies. An important hint arguing against the Trp-cage conformation being stabilized in the exendin-4 oligomer comes from Hudson et al., who found the C-terminus from Ser33–Ser39 being rather flexible in the oligomer, similar to statistical coils. They thus excluded the interaction of Trp25 with Pro37, which is one of the key characteristics for the Trp-cage conformation. The authors concluded from these data, that hydrophobic surface burial is apparently better accomplished by oligomerization than by formation of the Trp-cage [7]. Taken together, these data suggest that the NMR monomer structure of exendin-4 in 30% TFE is certainly not an ideal monomeric building block for modelling. However, it is undoubtedly well-suited concerning the dimension of the helical segment involved in inter-monomer interaction in our model. In addition, the geometry of the conserved hydrophobic moiety can be regarded as sufficiently similar in aqueous buffer and in 30% TFE, while the seven C-terminal amino acids certainly have a substantially higher degree of freedom in the exendin-4 oligomer than indicated in our structural model.

The latter might be of importance as these amino acids may play a role in shielding the conserved hydrophobic moiety. In this context, our C-terminally truncated Dual-Cex isoforms are highly instructive. Our data clearly evidence that further exposure of the conserved hydrophobic moiety by stepwise C-terminal truncation is sufficient for oligomerization of Dual-Cex (Figure 7). Assuming that the conformation of the conserved hydrophobic moiety is identical in exendin-4 and Dual-Cex, this implies that it must be at least partially shielded already in aqueous buffer. Interestingly, this exposure of the conserved hydrophobic moiety leads to a different kind of oligomerization than that which we observe for exendin-4, as it is less defined, suggesting constant addition of monomeric subunits with increasing concentration, likely via the presentation of hydrophobic interfaces to the surrounding solvent. While not as detailed as in our study, a role of the conserved hydrophobic moiety in the oligomerization of related peptide has been evidenced in earlier studies. In this context, the addition of the nine C-terminal amino acids of exendin-4 to glucagon results in dramatically improved solubility and stability of the highly aggregation-prone native glucagon peptide [2]. It is important to note that all exendin-4 derived assembly structures presented in our study are essentially stable, in contrast to glucagon. The GLP-2 analog teduglutide, which also exhibits the conserved hydrophobic moiety, self-assembles into defined pentamers, as shown by AUC [34]. Following NMR structures determined in water-TFE solutions, this cluster is solvent-exposed in the teduglutide monomer [35]. Self-assembly induces not only α-helical structure, but also a distinct near-UV CD signal around 300 nm, indicating a clustering of Trp residues, but not Phe residues in the oligomer [34]. This is different from our C-terminally truncated Dual-Cex variants in two aspects, first the rather defined nature of the teduglutide oligomers and second the apparent differences in the near-UV CD signals, which indicate involvement of both, Phe and Trp residues in the oligomerization of the C-terminally truncated Dual-Cex variants. Importantly, the effect of truncation of residues 34–39 of Dual-Cex on self-association and concomitant conformational changes of the conserved hydrophobic moiety is relatively low, while the effect of truncation of residues 30–39 is much more drastic. This suggests that a previously described interaction of Pro31 with Trp25 in aqueous buffer [7] is highly efficient in shielding Trp25, while contributions of farther C-terminally localized amino acids, likely Pro36–38, are less important. These considerations are in line with our structural hypothesis, which suggests that Trp25 of exendin-4 monomers do not interact with each other in the trimer, but rather with Pro31 and Pro36–38 of their own C-termini. Apparently, the actual impact of Pro36–38 on shielding of Trp25 might be overrated in our structural hypothesis due to the use of the TFE monomer structure with its stable Trp-cage conformation.

Additional information about the complicated interplay between the different forces driving association of Dual-Cex can be derived from different Dual-Cex isoforms conjugated to a palmitic acid. We have previously shown that palmitic acid fusion coerces Dual-Cex into an oligomeric state [4]. It is necessary to note that these assemblies differ from those of exendin-4 and might rather be imagined as quasi-micellar structures. Palmitic acid conjugation also leads to self-assembly of all Dual-Cex isoforms used in our study, but the resulting assembly structures are remarkably different from each other in terms of size and stability towards dilution (Figure 8). Apparently, an intact conserved hydrophobic moiety is mandatory for the formation of stable self-assemblies, as in contrast to Dual-Cex_C16, both palmitic acid conjugated Dual-Cex isoforms harboring the F22R mutation (Dual-Cex-C16_F22R and Dual-C16_F22R) show substantial dissociation upon dilution. Likely, overall hydrophobicity of the conserved moiety is no longer sufficient for stable self-assembly upon loss of Phe22, underlining the fundamental role of Phe22 interaction in the exendin-4 trimer, as suggested in our structural hypothesis. On the other hand, the exposure of the conserved hydrophobic moiety (Dual-C16) affects the molecular architecture of the fatty acid stabilized self-assemblies, resulting in dilution-stable oligomers with a larger number of monomeric subunits. The latter might be rationalized via the micelle-like nature of the palmitic acid enforced assemblies, allowing a higher number of C-terminally truncated monomeric subunits, a hypothesis which needs further research.

## 5. Conclusions

**Figure 11:**
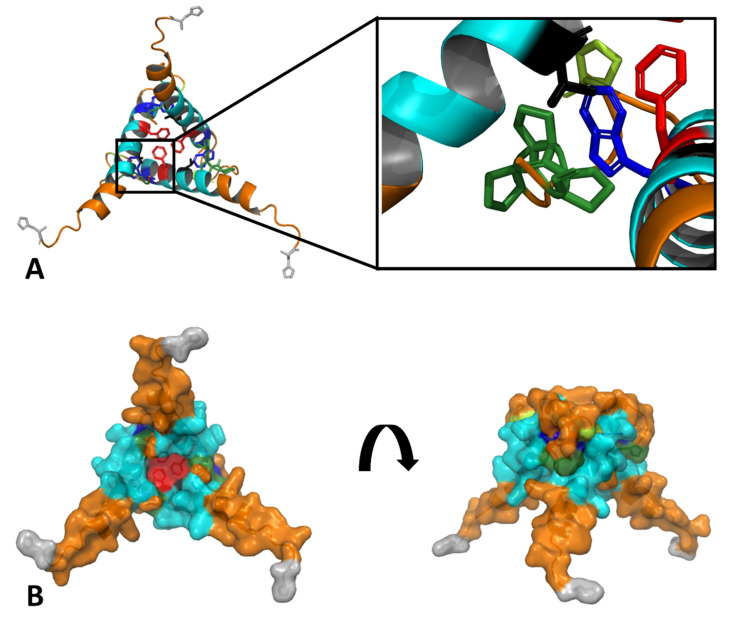
Structural hypothesis on the self-assembly of exendin-4 visualizing key interactions. The structural hypothesis introduced in Figure 2E was recolored, the hydrophobic moiety is shown in magnification (**A**). (**B**) shows the hypothesis in surface representation in different orientations for visualization of key interactions. N-termini are indicated in grey, the helical interface segment (AA13–26) is represented in cyan, Pro31 in light green and Pro 36–38 in dark green, respectively. Trp25 is shown in blue, Phe22 in red and Val19 in black colors. All other amino acids are shown in orange.

In summary, we here report the first published association equilibria of exendin-4 in aqueous solutions at different pH regimes directly measured by SLS. We utilize a structural hypothesis to rationalize experimentally validated key interactions in the smallest oligomer of exendin-4, the trimer at pH 7.4. Our structural hypothesis (Figure 11) pictures key interactions of exendin-4 monomers in the experimentally supported trimer, which are well-rationalized by integrating our own experimental data and findings reported by Hudson et al. [7]. These key interactions are helix–helix contacts of the helical interface segment (AA13–26) and a hydrophobic triangle constituted by the Phe22 residues of the three monomeric subunits. Val19 might function as an anchor in the N-terminus of the interacting helix-region. The Trp25 residues do not interact with each other in our structural hypothesis, but with the C-terminal Pro31 and Pro36–38 of their own monomers. This is in line with experimental data on Dual-Cex, which evidence the partial shielding of Trp25 by C-terminal amino acids of their own monomer, involving Pro31 and to a lesser degree also Pro36–38. Interestingly, our structural hypothesis also shows a close proximity of Pro31 and Pro36-38 with the helices of neighboring monomers in the trimer. Though such an interaction cannot be evidenced from our experimental data, it is attractive to speculate that such an additional interaction might further stabilize and could thus finally explain the rather defined nature of the exendin-4 trimer.

## Figures and Tables

**Table 1:** Specific absorbance and molecular mass of the peptides calculated with the ProtParam tool [19] from their amino acid sequences including the alkyl chain if present.

Peptide	Molecular Mass [Da]	A (280 nm, 1 cm Path Length, 1 mg/mL)
Exendin-4	4187.6	1.313
Dual-Cex	4234.6	1.299
Exendin-4_F22R	4212.6	1.306
Dual-Cex_F22R	4243.6	1.296
Dual-Cex_AA1-33	3728.0	1.475
Dual-Cex_AA1-29	3399.7	1.618
Dual-Cex-C16	4616.1	1.191
Dual-C16	3781.2	1.455
Dual-Cex-C16_F22R	4625.1	1.189
Dual-C16_F22R	3790.3	1.451

## Data Availability

Not applicable.

## Supplementary Materials

The following are available online at https://www.mdpi.com/article/10.3390/biom11091305/s1, Figure S1: Multiple sequence alignment of proglucagon-derived peptides, Figure S2: Titration of exendin-4 in PBS, pH 7.4 with TFE, Figure S3: Far-UV CD spectra of exendin-4 and Dual-Cex and the respective F22R mutants of both peptides, Figure S4: Temperature dependence of θ222 from far UV CD spectra for exendin-4 at 0.4 mM at pH 7.4, Figure S5: Compactness of exendin-4 in PBS, pH 7.4 and in PBS + 30% TFE, pH 7.4, Table S1: 1H-chemical shifts of Dual-Cex in 70% H_2_O/D_2_O (9:1), 100 mM acetate buffer, pH 4.6 and 30% TFE at 310 K, Table S2: 1H-chemical shifts of Dual-Cex in H_2_O/D_2_O (9:1), 100 mM acetate buffer, pH 4.6 at 310 K.
