# Supplementary material for: A Conserved Hydrophobic Moiety and Helix–Helix Interactions Drive the Self-Assembly of the Incretin Analog Exendin-4
Source: Biomolecules. 2021 Sep 3;11(9):1305. doi: 10.3390/biom11091305 (PMC8472270; doi:10.3390/biom11091305)
Supplement: Supplementary file 1 [file biomolecules-11-01305-s001.zip › biomolecules-1319800-supplementary.pdf]

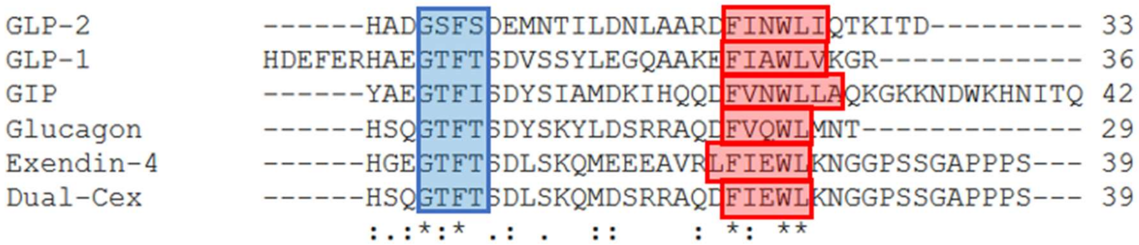

Figure S1. Multiple sequence alignment (Clustal O 1.3.4) of proglucagon-derived peptides. Conserved sequence clusters are highlighted. Red colors depict a cluster of conserved hydrophobic amino acids

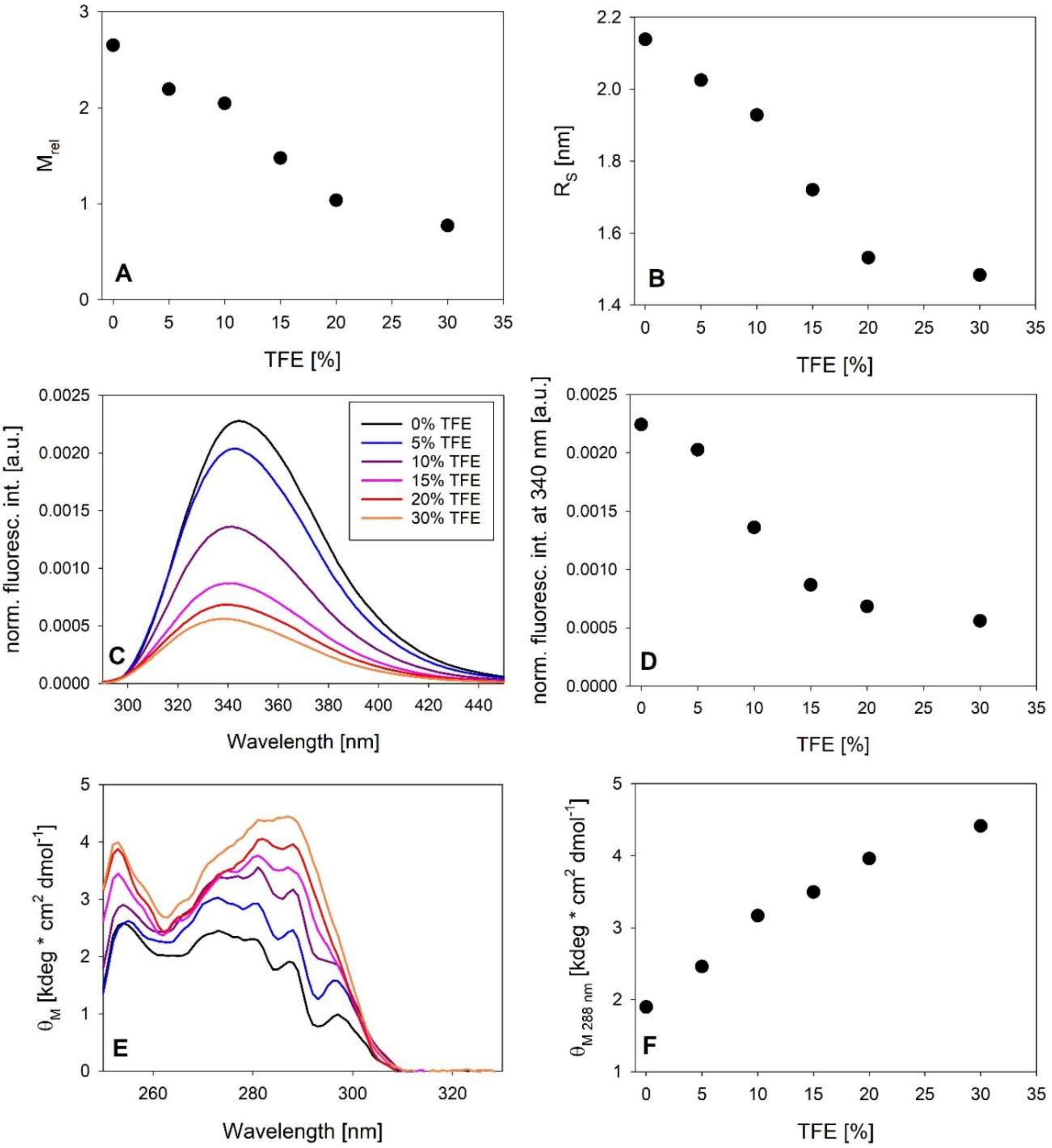

Figure S2. Titration of exendin-4 in PBS, pH 7.4 with TFE. (**A**)and (**B**) show relative molecular masses M_{rel} (**A**) and Stokes radii R_S (**B**) of 0.3 mM exendin-4 with increasing concentrations of TFE measured at 23 °C. Trp fluorescence (**C**) and near-UV CD (**E**) spectra of 0.1 mM exendin-4 were measured with increasing concentrations of TFE. Fluorescence intensity at 340 nm and molar ellipticity at 288 nm were plotted as a function of

TFE concentration in (**D)** and (**F)**, respectively. Fluorescence intensity is shown normalized to peptide concentration and solvent contribution.

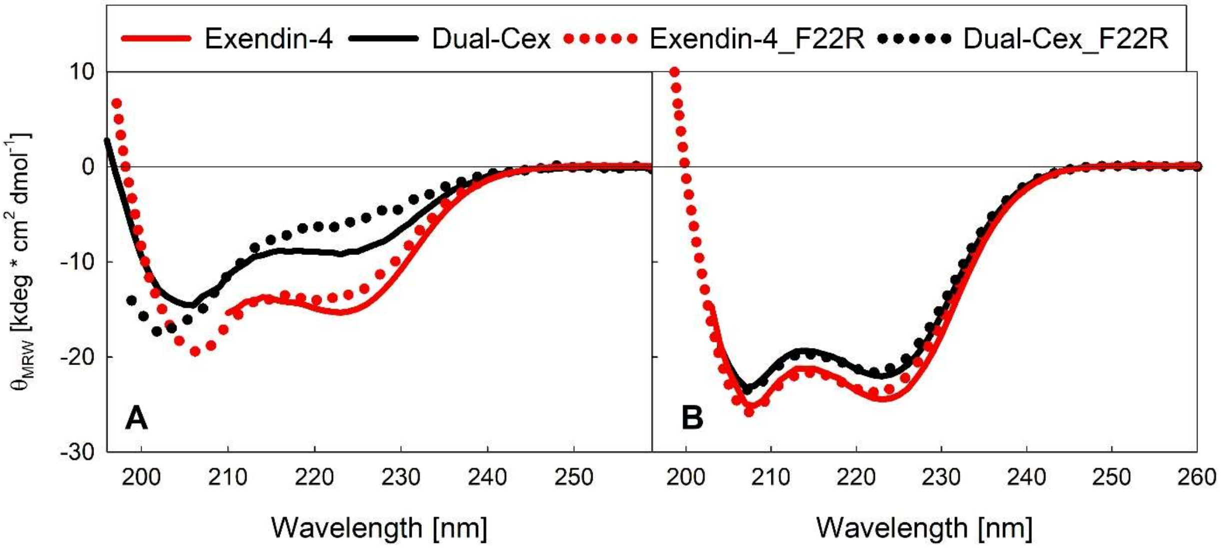

Figure S3. Far-UV CD spectra of exendin-4 and Dual-Cex and the respective F22R mutants of both peptides were measured in PBS (**A**) and in 30% TFE (**B**) at pH 7.4 at concentrations < 0.1 mM which grant a monomeric state of all peptides

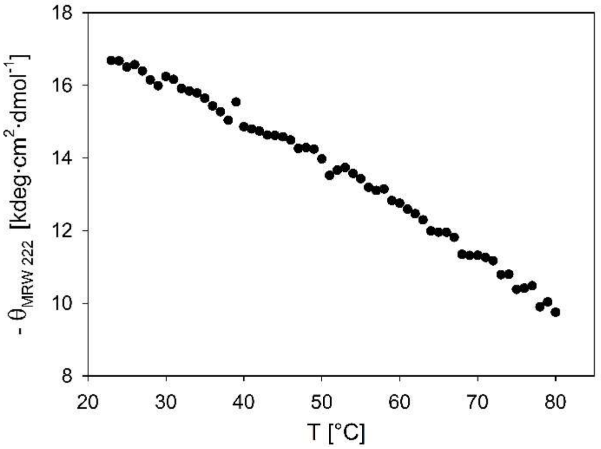

Figure S4. Temperature dependence of θ_{222} from far UV CD spectra for exendin-4 at 0.4 mM at pH 7.4

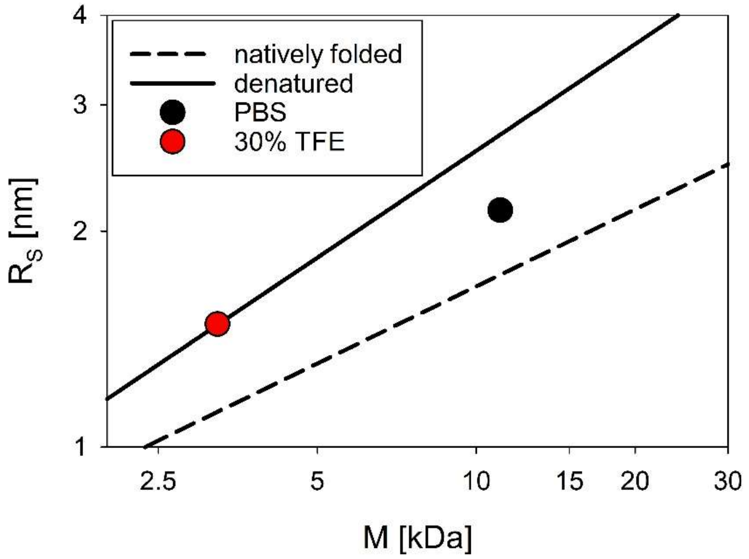

Figure S5. Compactness of exendin-4 in PBS, pH 7.4 (black) and in PBS + 30% TFE, pH 7.4 (red). Compactness of ~0.4 mM exendin-4 and at 23 °C, visualized using scaling laws of the type $R_S = a \cdot M^b$. Reference data sets reporting on the average compactness of natively folded (dashed line) and denatured (solid line) proteins are shown for comparison.

Table S1. ^{1}H-chemical shifts of Dual-Cex in 70% H_2O/D_2O (9:1), 100 mM acetate buffer, pH 4.6 and 30% TFE at 310 K[1].

No.	Residue	NH	Hα	Hβ	Others
1	His	-			δ: 7.28, ε: 8.27
2	Ser	Broad	4.58	3.96/3.92	
3	Gln	8.70	4.45	2.21/2.08	γ: 2.44, NH2: 7.43/6.72
4	Gly	8.40	4.03		
5	Thr	7.90	4.37	4.22	γ: 1.17
6	Phe	8.18	4.70	3.22/3.15	δ: 7.30, ε: 7.36, ζ: 7.30
7	Thr	7.87	4.28	4.28	γ: 1.23
8	Ser	8.10	4.42	4.01/3.92	
9	Asp	8.29	4.63	2.78/2.73	
10	Leu	8.12	4.20	1.67	γ: 1.67, δ: 0.94, δ′: 0.89
11	Ser	8.14	4.22	4.03/3.99	
12	Lys	7.78	4.29	1.98/1.95	γ: 1.60/1.50, δ: 1.75, ε: 3.03
13	Gln	8.01	4.24	2.22/2.18	γ: 2.45, NH2: 7.27/6.63
14	Met	8.25	4.36	2.15	γ: 2.68/2.59, ε: 2.05
15	Asp	8.22	4.59	2.86/2.78	
16	Ser	8.18	4.32	4.08/4.02	
17	Arg	8.08	4.24	2.06/2.00	γ: 1.79, δ: 3.27, ε: 7.51

[1] Proton chemical shifts are referenced to sodium-3-(Trimethylsilyl)propionate-2,2,3,3-d_4.

18	Arg	8.12	4.25	1.99	γ: 1.86/1.72, δ: 3.26, ε: 7.26
19	Ala	8.05	4.28	1.58	
20	Gln	8.145	4.08	2.27	γ: 2.58/2.49, NH2: 7.39/6.61
21	Asp	8.32	4.54	2.96/2.68	
22	Phe	8.26	4.34	3.39/3.30	δ: 7.22, ε: 7.34, ζ: 7.38
23	Ile	8.51	3.76	2.14	β-Me: 1.02, γ: 1.92/1.43, δ: 0.96
24	Glu	8.32	4.03	2.29/2.22	γ: 2.51
25	Trp	8.10	4.30	3.76/3.26	H1: 9.76, H2: 7.18, H4: 7.28, H5: 7.06, H6: 7.13, H7: 7.27
26	Leu	8.55	3.40	1.95/1.42	γ: 1.64, δ: 0.85, δ’: 0.80
27	Lys	8.84	3.93	1.97/1.92	γ: 1.68/1.47, δ: 1.68, ε: 2.96
28	Asn	7.58	4.76	3.00/2.78	NH2: 7.62/6.88
29	Gly	7.66	4.19/3.55		
30	Gly	8.24	2.77/1.42		
31	Pro	-	4.53	2.46/2.03	γ: 2.08/2.03, δ: 3.65/3.05
32	Ser	7.67	4.43	3.97/3.92	
33	Ser	8.04	4.33	3.99/3.77	
34	Gly	7.84	4.29/3.81		
35	Ala	8.06	4.89	1.49	
36	Pro	-	4.63	2.21/1.87	γ: 2.02, δ: 3.85/3.69
37	Pro	-	3.13	1.47/1.05	γ: 1.80/1.62, δ: 3.45

38	Pro	-	4.36	2.24/1.96	γ: 1.89, δ: 3.16/3.02
39	Ser	7.93	4.34	3.88/3.80	NH2: 7.41/6.93

Table S2. ¹H-chemical shifts of Dual-Cex in H_2O/D_2O (9:1), 100 mM acetate buffer, pH 4.6 at 310 K[2].

No.	residue	NH	Hα	Hβ	Others
1	His		4.33	3.36	δ: 7.34, ε: 8.47
2	Ser		4.54	3.88	
3	Gln	8.69	4.42	2.16/2.03	γ: 2.41, NH2: 7.48/6.83
4	Gly	8.41	3.98		
5	Thr	7.95	4.31	4.12	γ: 1.12
6	Phe	8.31	4.73	3.17/3.03	δ: 7.25, ε: 7.33, ζ: 7.28
7	Thr	8.00	4.33	4.20	γ: 1.17
8	Ser	8.21	4.42	3.91/3.85	
9	Asp	8.32	4.62	2.76/2.66	
10	Leu	8.19	4.26	1.65	γ: 1.65, δ: 0.92, δ′: 0.85
11	Ser	8.21	4.31	3.95/3.90	
12	Lys	7.92	4.30	1.89/1.78	γ: 1.47/1.41, δ: 1.68, ε: 2.99
13	Gln	8.06	4.29	2.13/2.01	γ: 2.37, NH2: 7.44/6.78
14	Met	8.21	4.41	2.07/2.02	γ: 2.58/2.52, ε: 2.03
15	Asp	8.22	4.61	2.75	
16	Ser	8.21	4.33	3.96/3.91	
17	Arg	8.19	4.26	1.91/1.85	γ: 1.67/1.62, δ: 3.19, ε: 7.37

[2] Proton chemical shifts are referenced to sodium-3-(Trimethylsilyl)propionate-2,2,3,3-d_4.

18	Arg	8.09	4.28	1.89/1.83	γ: 1.70/1.64, δ: 3.18, ε: 7.26
19	Ala	8.18	4.26	1.48	
20	Gln	8.25	4.18	2.12	γ: 2.43, NH2: 7.52/6.79
21	Asp	8.24	4.52	2.77/2.67	
22	Phe	8.14	4.33	3.19/3.14	δ: 7.17, ε: 7.32, ζ: 7.34
23	Ile	7.97	3.79	1.98	β-Me: 0.88, γ: 1.65/1.30, δ: 0.89
24	Glu	8.12	4.07	2.09	γ: 2.39
25	Trp	7.97	4.38	3.45/3.24	H1: 9.88, H2: 7.18, H4: 7.32, H5: 7.07, H6: 7.17, H7: 7.36
26	Leu	8.16	3.68	1.70/1.46	γ: 1.53, δ: 0.84, δ′: 0.80
27	Lys	8.11	4.05	1.85	γ: 1.52/1.40, δ: 1.65, ε: 2.94
28	Asn	7.85	4.74	2.91/2.75	NH2: 7.54/6.88
29	Gly	7.84	4.03/3.71		
30	Gly	7.98	3.33/2.75		
31	Pro	-	4.46	2.34/1.98	γ: 2.00, δ: 3.58/3.22
32	Ser	8.06	4.45	3.92/3.89	
33	Ser	8.12	4.41	3.94/3.80	
34	Gly	8.11	4.09/3.90		
35	Ala	7.96	4.67	1.38	
36	Pro	-	4.61	2.22/1.83	γ: 2.00, δ: 3.80/3.62

37	Pro	-	3.93	1.69	γ: 1.90/1.86, δ: 3.65/3.51
38	Pro	-	4.38	2.26/1.90	γ: 1.90, δ: 3.45/3.29
39	Ser	8.16	4.35	3.87/3.82	NH2: 7.50/7.08
